# A Rare Case of Primary Thyroid Lymphoma

**DOI:** 10.7759/cureus.109116

**Published:** 2026-05-18

**Authors:** David Zitser, Amanda Frugoli, Lynn Kong, Tina Nikoomanesh, Nugdeep Singh

**Affiliations:** 1 Graduate Medical Education, Community Memorial Healthcare, Ventura, USA; 2 Pacific Inpatient Physicians, Community Memorial Hospital, Ventura, USA; 3 Graduate Medical Education, Community Memorial Hospital, Ventura, USA; 4 Oncology, Ventura County Hematology Oncology Specialists, Ventura, USA

**Keywords:** large b cell lymphoma, non-pth related hypercalcemia of malignancy, primary thyroid lymphoma, primary thyroid lymphoma (ptl), rare thyroid malignancy, substernal goiter, thyroid cancer

## Abstract

Primary thyroid lymphomas (PTL) are a unique malignancy that arises from within the thyroid gland and is not typically considered in the differential diagnosis of thyroid cancers. PTL is strongly associated with underlying autoimmune thyroiditis; despite this association, there are no screening guidelines. Management of PTL involves chemotherapy, contrasting with the surgical and radioactive iodine therapies employed in differentiated thyroid cancers.

We report a case of a 68-year-old male with a history of hypothyroidism and multiple comorbidities who presented with a rapidly enlarging, painless goiter, dysphagia, and symptoms of hypothyroidism, including fatigue and constipation. Laboratory evaluation revealed severe hypothyroidism and marked non-PTH-mediated hypercalcemia with associated acute renal failure. Imaging of the neck identified a large circumferential mass extending below the clavicles. Ultrasound-guided biopsy confirmed diffuse large B-cell lymphoma (DLBCL). The patient was treated initially for myxedema and hypercalcemia and subsequently initiated on R-CHOP (Rituximab, Cyclophosphamide, Doxorubicin, Vincristine, and Prednisone) chemotherapy for stage IIE diffuse large B-cell lymphoma.

This case highlights an uncommon presentation of symptoms with confounding hypothyroidism. We also note that this is an uncommon malignancy in males. The case vignette emphasizes the importance of distinguishing PTL from other thyroid malignancies, given differences in prognosis and treatment. PTL is a rare but clinically significant malignancy requiring prompt recognition and tailored treatment.

## Introduction

Primary thyroid lymphoma (PTL) can be mistaken for primary thyroid cancer due to its location and presentation as an enlarging neck mass or goiter [1-3]. PTLs are distinct cancers that present as masses within the thyroid gland [1,4,5]. They are nearly always non-Hodgkin lymphomas (NHLs) [6]. PTLs are more commonly diffuse large B-cell, but can include marginal zone B-cell lymphoma/mucosa-associated lymphoid tissue (MALT) and follicular lymphoma [5,6]. PTL is distinctly different from thyroid cancer, which refers to malignant tumors arising from the thyroid gland, including differentiated thyroid cancers (papillary, follicular), medullary thyroid carcinoma, and anaplastic thyroid carcinoma [4,3,7].

PTL comprises less than 3% of extranodal lymphoma and less than 5% of all thyroid malignancies [7]. It has an estimated annual incidence of 2 per 1 million individuals [7,8]. Similarly to PTC, it has a predisposition for women, with a median age of presentation of 65 years old. The female-to-male ratio is 4:1 [3,7]. The most common types of PTL are non-Hodgkin lymphomas of B-cell origin. Diffuse large B-cell lymphoma (DLBCL) accounts for approximately 60% to 70% of cases, and follicular lymphoma accounts for 10% of cases [8-10]. Patients with Hashimoto’s thyroiditis are especially at risk. In fact, 60-90% of PTLs occur in patients with thyroiditis, with cancer developing years after the diagnosis of hypothyroidism [7,8]. Although PTL is classified within the broader group of lymphomas, its risk factors differ from those of other lymphoma subtypes [8]. Overall, PTL has a median overall survival of 9.3 years and a 5-year overall survival of 66%, with DLBCL having the lowest disease-specific survival rate of 75% and a nearly 5 times likelihood of death compared with MALT lymphoma [8-10].

The clinical presentation of PTL varies by lymphoma subtype, with DLBCL being particularly aggressive compared to other lymphomas, such as MALT and follicular lymphoma [10]. Similar to ATC, DLBCL patients often present with obstructive symptoms like dyspnea, dysphagia, edema of the face secondary to compression of the neck veins, and hoarseness of the voice secondary to compression of the recurrent laryngeal nerve [10]. In addition, systemic symptoms or "B symptoms", such as fever, night sweats, and weight loss, are often present. Signs of hypothyroidism, such as cold intolerance, weight gain, and myxedema, are typically secondary to underlying Hashimoto’s rather than thyroid follicle destruction, which is more likely to cause transient hyperthyroidism [7]. Due to its location, biopsy and histopathologic evaluation are always necessary to differentiate DLBCL from other thyroid diseases. DLBCL is characterized by a diffuse proliferation of large, mature B cells, often larger than twice the normal size of lymphocytes [6]. On pathology, DLBCL of the thyroid would show diffuse infiltrates destroying thyroid follicles, large cells with moderate amphophilic cytoplasm, vesicular nuclei, and prominent nucleoli [6]. For staging, the National Comprehensive Cancer Network (NCCN) adopts the Lugano modification of the Ann Arbor staging system [10]. Stage I refers to one node or a group of adjacent nodes, stage II refers to two or more nodal groups on the same side of the diaphragm, stage III refers to nodes on both sides of the diaphragm, and stage IV refers to additional non-contiguous extralymphatic involvement [10-12]. The stage-modified international prognostic index (smIPI) is commonly used to predict outcomes in patients with limited DLBCL, in which it attributes a point for age over 60, Ann Arbor Stage of III and IV, an Eastern Cooperative Oncology Group (ECOG) performance score of over 1, lactate dehydrogenase (LDH) above the normal level, and involvement of an extranodal site [11-13].

We present this case vignette of primary thyroid lymphoma to bring awareness of cancers that present as rapidly enlarging goiters. 

## Case presentation

A 68-year-old male with type 2 diabetes with associated chronic kidney disease stage 3, hypothyroidism, and bipolar disease presented to the emergency room with complaints of non-painful circumferential neck enlargement and associated dysphagia for the last 3 weeks. He reported gradual worsening of dysphagia but was still able to consume liquids and solids. He endorsed associated symptoms of lethargy, brain fog, difficulty walking, chills, decreased appetite, and weight loss. Review of systems was negative for neck pain, difficulty breathing, nausea, vomiting, fever, chest pain, or palpitations.

Medications included quetiapine by mouth 300 mg twice daily, gabapentin by mouth 300 mg three times daily, and aspirin 81 mg daily. He had recently self-discontinued metformin and levothyroxine for unclear reasons.

His exam was notable for a frail-appearing man wearing a cap, sweater, and heavy jacket despite the summer weather. His neck exam showed marked thyromegaly extending from his jawline down to the sternum. His thyroid was firm, rubbery, and nontender to palpation. The trachea was not palpable due to the goiter. The cardiovascular exam showed a regular rate, regular rhythm, with a systolic murmur noted loudest in the axilla. No lower extremity edema or jugular vein distention was noted. The skin showed pallor and was cool to the touch.

Table 1 lists the laboratory values on presentation.

**Table 1:** Laboratory results on presentation Laboratory results demonstrate a significant elevation in TSH consistent with severe hypothyroidism. The laboratory also demonstrates severe non-PTH-mediated hypercalcemia with associated acute renal failure. Elevations are noted in PTHrP and calcitriol.

Test	Result & Units	Reference Range
Thyroid Stimulating Hormone (TSH)	92 µIU/mL	0.4 – 4.0 µIU/mL
Thyroxine (T4)	0.6 µg/dL	4.5 – 11.2 µg/dL
Free Thyroxine Index	0.20%	1.0 – 4.5 %
T3 Uptake	35%	24 – 39 %
Thyroglobulin (Thyroid)	—	—
Sodium (Na)	140 mmol/L	135 – 145 mmol/L
Potassium (K)	2.8 mmol/L	3.5 – 5.0 mmol/L
Chloride (Cl)	92 mmol/L	96 – 106 mmol/L
Bicarbonate (Bicarb)	30 mmol/L	22 – 28 mmol/L
Blood Urea Nitrogen	26 mg/dL	7 – 20 mg/dL
Creatinine (Cr)	2.69 mg/dL	0.6 – 1.3 mg/dL
Calcium (Ca)	15.9 mg/dL	8.5 – 10.5 mg/dL
Parathyroid Hormone (PTH)	5 pg/mL	10 – 65 pg/mL
Vitamin D (25-OH D)	23 ng/mL	20 – 50 ng/mL
1,25-dihydroxycholecalciferol (25(OH)2D)	197 pg/mL	18–64 pg/mL (males)
Parathyroid hormone-related peptide (PTHrP)	3.2 pmol/L	<2.0 pmol/L

Imaging with a CT scan without contrast of the neck demonstrated bilateral soft-tissue masses in the cervical region, with the left greater than the right. In addition, adenopathy was noted in the posterior cervical triangles. CT scan of the chest/abdomen/pelvis showed the inferior margin of the mass extending to the superior mediastinum to the level of the arch, but no additional adenopathy or hepatosplenomegaly (Figure 1 and Figure 2).

**Figure 1:**
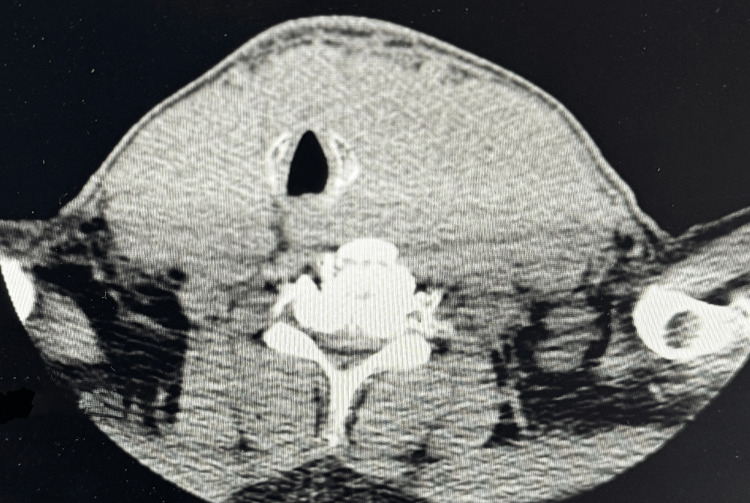
Coronal CT imaging demonstrating a large neck mass with some displacement of the airway In this coronal CT image, the trachea is slightly displaced with evidence of a paratracheal circumferential mass that extends from the anterior neck into the superior chest.

**Figure 2:**
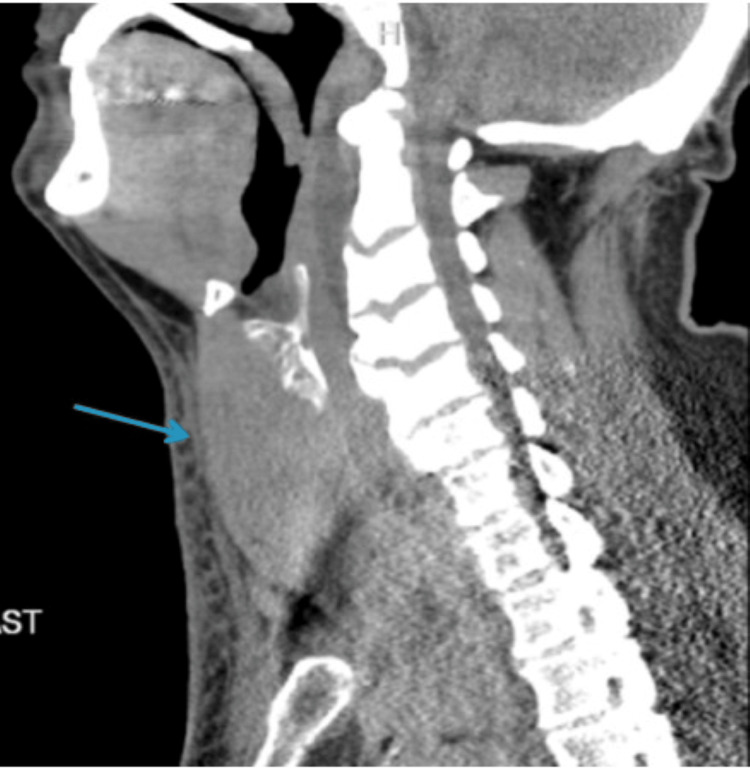
Non-contrast enhanced CT of the neck demonstrating an enlarged thyroid gland (blue arrow) Sagittal CT images demonstrate a large anterior neck mass in the region of the thyroid bed.

Endocrinology was consulted, and empiric treatment for myxedema was started with intravenous levothyroxine and oral steroids due to significantly elevated TSH in the setting of recent levothyroxine cessation with a rapidly enlarging goiter. Nephrology was consulted for acute renal failure and severe hypercalcemia. Hypercalcemia with a concentration peak of 15.5 mg and suppressed PTH raised concern for malignancy. This non-PTH-mediated hypercalcemia was symptomatic and a worrisome symptom for a disease not related to hypothyroidism. When combined with weight loss, rapidly enlarging goiter, and possible fevers, this was concerning for malignancy, but not consistent with typical thyroid cancer presentations. Acute hypercalcemia was treated with IV fluids at 200 cc/hr and forced diuresis w/ IV furosemide. He was given calcitonin at 0.4 units/kg once. Bisphosphonate therapy was contraindicated due to a glomerular filtration rate (GFR) of under 30.

He underwent a US-guided biopsy, which demonstrated a large B-cell lymphoma with fluorescence in situ hybridization (FISH) analysis confirming diffuse B-cell lymphoma, with a non-germinal center type and a high proliferation rate (90%) (CD10-, CD23-, BCL6+, MUM1+, BCL-2-, cMYC-) (Figures 3, 4). A bone marrow biopsy for staging showed a normocellular bone marrow and no evidence of lymphoma. This is consistent with B-cell-lymphoma stage IIE disease. 

**Figure 3:**
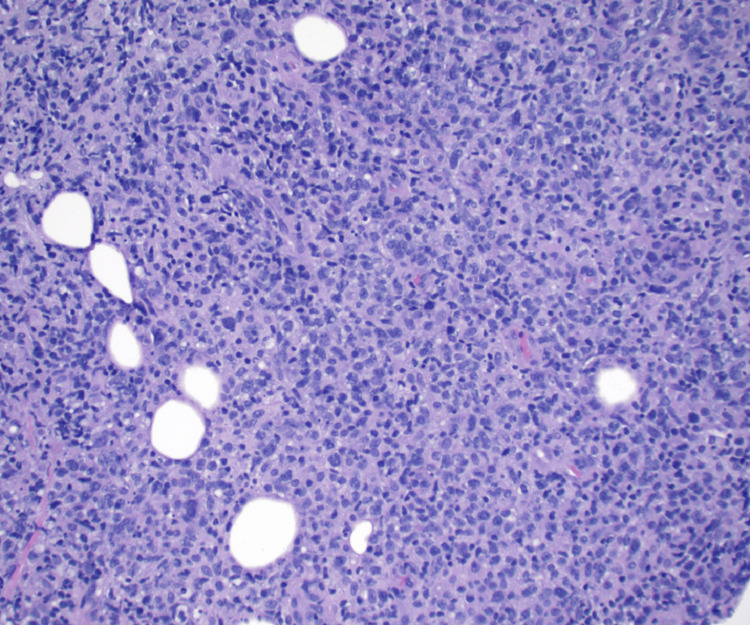
Core biopsy from the neck mass showing sheets of malignant lymphoid cells with nuclear pleomorphism and numerous mitotic figures ( H&E; 200X magnification)

**Figure 4:**
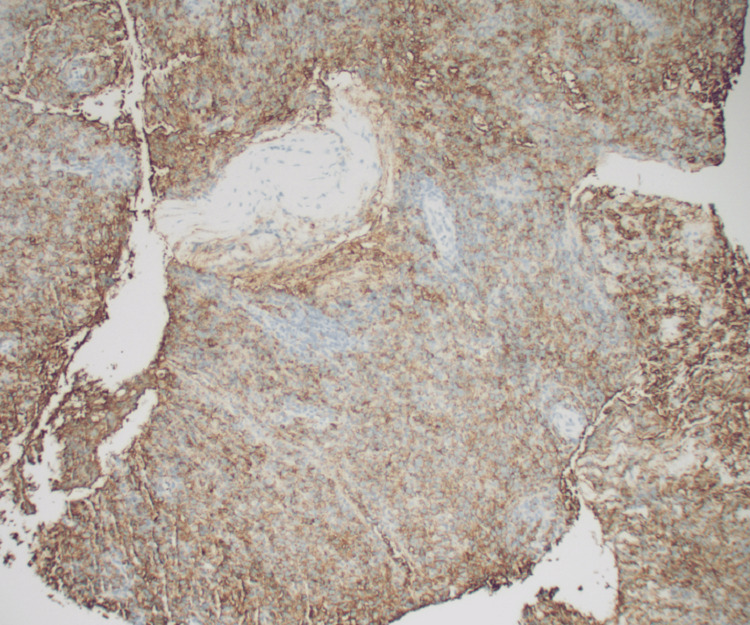
Malignant cells are diffusely positive for CD20 by immunohistochemistry, compatible with B-cell lymphoma (100X magnification)

He responded to IV hydration with gradual improvement in his renal function and resolution of hypercalcemia. He started systemic chemotherapy with R-CHOP, consisting of Rituximab, Cyclophosphamide, Doxorubicin, Vincristine, and Prednisone, while inpatient, with plans to complete six cycles of treatment as an outpatient. A review of the timeline of events is given in Figure 5. 

**Figure 5:**
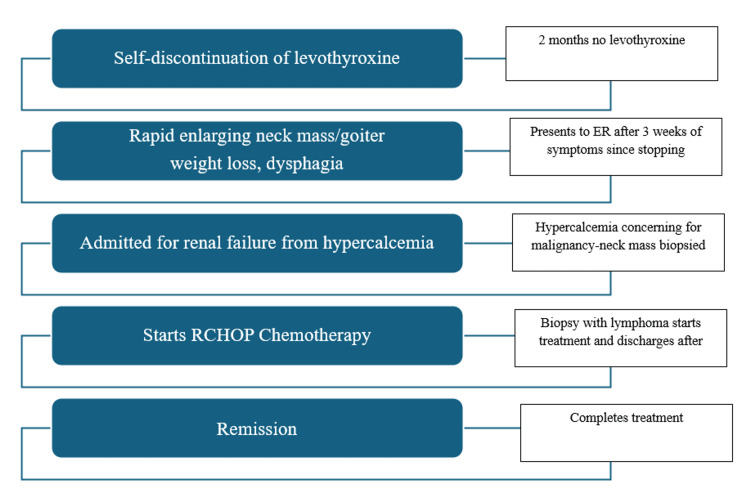
Timeline of events

## Discussion

This is an interesting case, as our patient is a male (most typically, PTL is seen in females as discussed above) who presented with significant hypothyroid symptoms after self-discontinuation of levothyroxine, a rapidly enlarging neck goiter with obstruction symptoms, and non-PTH-mediated hypercalcemia. Initially, a review of this case presentation would have led to a common diagnosis, assuming it was a goiter from Hashimoto's thyroiditis in the setting of self-discontinuation of levothyroxine. However, it is crucial to identify these subtle nuances - the rapid growth and compressive symptoms, non-PTH-dependent hypercalcemia, and history of Hashimoto’s disease - all hinting at something far more concerning. Once this is understood, broadening the differential to include malignancy and pursuing the next steps in imaging are key to the correct diagnosis. It is also important to recognize that thyroid cancers may present with a neck mass but are not associated with hypercalcemia. 

Hypercalcemia, in this case, presented with suppressed PTH. Hypercalcemia in non-Hodgkin lymphoma is most common in diffuse large B-cell lymphoma [14]. The mechanism is primarily driven by calcitriol over PTHrP, but can be heterogeneous [13,14]. Hypercalcemia presentation can occur in 30% of patients with lymphoma and denotes overall worse outcomes [13,14]. Severe hypercalcemia is the most likely cause for his presentation with acute renal failure that improved to his baseline with IVF and forced diuresis. 

In our case, we hypothesize that the patient’s risk factors for a thyroid lymphoma were related to known Hashimoto’s thyroiditis. Patients with Hashimoto’s thyroiditis have a significantly increased risk (40-80x) of developing thyroid lymphoma, as chronic inflammation can result in lymphoid growth and malignant transformation [6,8]. Our case, although rare, does represent the most common primary thyroid lymphoma subtype, diffuse large B-cell lymphoma (50-90%) [5,6]. Our patient's histology and immunoprofile (CD10-, BCL6+, MUM1+) are consistent with diffuse large B-cell lymphoma, non-germinal center type. Molecular studies for our patient are negative for BCL6, BCL2, and MYC rearrangements and for MYC amplification, ruling out “double/triple hit” lymphoma [5]. This is an important distinction, as these genetic arrangements are associated with aggressive, treatment-resistant disease with significantly reduced survival [5]. We suspect his presenting symptoms were a combination of overt hypothyroidism, hypercalcemia of malignancy related to lymphoma, and mass effect from PTL. It is possible his weight loss and reported chills were "B" symptoms associated with lymphoma, instead of being related to dysphagia from an enlarging goiter and reduced metabolism from hypothyroidism. 

In accordance with the NCCN, our patient was diagnosed with stage IIE disease with initial therapy of R-CHOP, with the first cycle started during his hospitalization due to the severity of hypercalcemia and obstructive symptoms [11,13]. He completed treatment and is in remission at the time of publication.

## Conclusions

Primary thyroid lymphomas arise from the thyroid bed, with a majority being diffuse large B-cell lymphoma. Chronic inflammation from Hashimoto's thyroiditis is a significant risk factor. Patients with an enlarging neck or goiter, with or without obstructive symptoms, should undergo further evaluation. Key clinical “red flags” observed in this patient included rapid growth of goiter, obstructive symptoms, and non-PTH-dependent hypercalcemia. PTL presentation can be confused with primary thyroid cancer due to its location. Histopathology is necessary to distinguish this from classic thyroid malignancies. Primary thyroid lymphoma has a significantly different treatment course from classic thyroid cancers that involves chemotherapy instead of surgical and radioactive iodine therapies. More studies are necessary to determine if cancer screening is indicated in patients with autoimmune thyroid disease.
